# Mitochondrial transcripts and associated heteroplasmies of *Ancistrus* spp. (Siluriformes: Loricariidae)

**DOI:** 10.1016/j.dib.2015.09.010

**Published:** 2015-10-22

**Authors:** Daniel A. Moreira, Carolina Furtado, Thiago E. Parente

**Affiliations:** a Laboratório de Toxicologia Ambiental, DCB, Escola Nacional de Saúde Pública – ENSP, Fundação Oswaldo Cruz – FIOCRUZ, Rio de Janeiro, Brasil; b Divisão de Genética, Instituto Nacional do Cancer – INCA, Rio de Janeiro, Brasil

## Abstract

This data-set complements our paper entitled “The use of transcriptomic next-generation sequencing data to assembly mitochondrial genomes of *Ancistrus* spp. (Loricariidae)” [6]. Here, we present the nucleotide sequences of each transcript used for mitogenomes assembly, as well as tables presenting the location of each transcript in the mitogenomes; the frequency, location and codon position of the detected heteroplasmic sites; and the start/stop codons usage, UTR, CDS and poliA-tail length for each protein coding gene. Readers are referred to the paper cited above for data interpretation and discussion.

## Specifications table

**Table:** 

Subject area	*Biology*
More specific subject area	*Biodiversity*
Type of data	*Table, text (.fasta) file*
How data was acquired	*Illumina HiSeq2500*
Data format	*Analyzed*
Experimental factors	*Raw reads were assembled by Trinity*
Experimental features	*Mitochondrial transcripts were identified by BLASTN against two other Loricariidae mitogenomes*
Data source location	*Fishes were donated by a fish exporter and sampled in the Amazon region*
Data accessibility	*Genbank* KP960567, KP960568, KP960569

## Value of the data

• These are the first sequences of long mitochondrial transcripts from *Ancistrus* spp.
• This data will aid primer design for other Loricariidae species in phylogenetic studies.
• Heteroplasmies position and frequency can be compared to other species.
• Start/stop codons usage, UTR, CDS and poliA-tail length for protein coding gene are described.

## 1 Data

Nucleotide sequences of each transcript used to assemble the mitogenomes of three *Ancistrus* spp. fish described by Moreira et al. [6] are available in fasta format (supplemental material). Table 1 shows the number and the length of each of those mitochondrial transcripts, as well as the maximum number of supporting reads per individual nucleotide and the total sum of supporting reads for each transcript. The position of heteroplasmic sites is shown at Table 2, along with the read counts of each nucleotide, the gene category and the codon position of each heteroplasmy. Specifically for protein-coding genes, Table 3 shows the lengths of 5′UTR, 3′UTR, ORF and Poly-A tail. In addition, Table 3 also shows the initiation and the termination codon, as well as the number of Adenines added to complete the stop codons, if needed.

## 2 Experimental design, materials and methods

Fish sampling, RNA extraction, Illumina library preparation and sequencing, raw reads processing and transcriprtome assemble are described elsewhere [6]. Each transcriptome was subjected to a BLASTN against the complete mitogenome of the closest related species, whose mitogenome is publically available, *Pterygoplichthys disjunctivus* (GI: 339506171) [7]. The transcripts aligned to the reference mitogenome were used for the mitogenomes assembly. The selected transcripts were edited according to the information of strand orientation given by the BLASTN result, and aligned by SeaView using the built-in CLUSTAL alignment algorithm and the reference mitogenome [2]. A CONTIG sequence was generated using the sequence information of just the transcripts of each individual fish. The sequence of the CONTIG was then manually checked for inconsistencies and gaps. The mitogenomes were annotated using the web-based services MitoFish and MITOS [1], [3] and features in protein-coding genes were analyzed according to Temperley et al. [9]. In order to estimate the support of each base in the mitogenomes, Bowtie v. 1.0.0 was used to align the reads of each fish on its own assembled mitogenome, and this mapping was viewed using the Integrated Genome Viewer (IGV) or the Tablet [10], [4], [5], [8]. Heteroplasmic sites were detected using IGV, setting the software to show positions in which the frequency of the second most frequent base was equal to or higher than 10% and the total reads number were higher than 100.

## Footnotes

Appendix A Supplementary data associated with this article can be found in the online version at doi:10.1016/j.dib.2015.09.010.
