# Supplementary material for: Mitochondrial transcripts and associated heteroplasmies of Ancistrus spp. (Siluriformes: Loricariidae)
Source: Data Brief. 2015 Oct 22;5:513–5. doi: 10.1016/j.dib.2015.09.010 (PMC4631843; doi:10.1016/j.dib.2015.09.010)
Supplement: Supplementary file 4 — Supplementary material [file mmc4.pdf]

Transcripts used	Position	Maximum number of support reads	Total read count
***Ancistrus* sp. #1 -** KP960569			
TR3967|c0_g1_i2_rc	17 - 2763	6790	54114
TR20743|c0_g1_i1_rc	2787 - 3813	10197	61326
TR20743|c1_g1_i1	3793 - 5071	3853	21244
TR953|c0_g1_i1_rc	5010 - 5452	945	1567
TR5060|c0_g2_i1	5453 - 7083	61299	593440
TR31091|c0_g1_i1	7040 - 7859	51623	182841
TR35939|c0_g1_i1	7805 - 8776	18038	72947
TR41077|c0_g1_i1_rc	8786 - 9560	84731	273214
TR41177|c0_g1_i1_rc	9504 - 9981	4083	6687
TR25681|c0_g1_i1_rc	9927 - 11721	4979	45925
TR25859|c0_g1_i1_rc	11824 - 15484	16861	95552
TR2173|c1_g1_i1_rc	15442 - 15724	141	144
TR2173|c0_g1_i1_rc	15707 - 16570	414	1536
**Total mitochondrial read count**			1410537
**% of total reads**			2.61%
***Ancistrus* sp. #2a** - KP960568			
TR29785|c0_g2_i1_rc	81 - 1008	2671	13355
TR29523|c0_g1_i1_rc	1044 - 2764	3824	24740
TR39048|c0_g1_i1_rc	2788 - 3814	10851	62035
TR6740|c0_g2_i1	3761 - 5072	6120	30330
TR6740|c0_g1_i1	5083 - 7085	61768	481349
TR20603|c0_g1_i1_rc	7031 - 7861	63235	182244
TR34530|c0_g1_i1	7806 - 8778	18296	70619
TR20428|c0_g1_i1_rc	8788 - 9562	54103	164505
TR4098|c0_g1_i1_rc	9538 - 9983	10742	17917
TR4098|c1_g1_i1	9960 - 11723	3024	27416
TR28461|c0_g1_i1_rc	11674 - 11908	7	8
TR34635|c0_g1_i1_rc	11946 - 15486	14952	86830
**Total mitochondrial read count**			1161348
**% of total reads**			1.79%
***Ancistrus* sp. #2b** - KP960567			
TR5076|c0_g1_i1	81 - 2768	4664	47386
TR5076|c1_g1_i1_rc	2745 - 5072	6896	72964
TR1889|c0_g1_i1_rc	5012 - 7085	39382	292735
TR31601|c0_g1_i1	7024 - 7861	28342	87015
TR31601|c1_g1_i1_rc	7838 - 9562	33598	137522
TR31601|c2_g1_i1_rc	9539 - 15486	5955	74600
TR27155|c0_g1_i1_rc	15438 - 15724	20	27
**Total mitochondrial read count**			712249
**% of total reads**			0.83%
