# Supplementary material for: Mitochondrial transcripts and associated heteroplasmies of Ancistrus spp. (Siluriformes: Loricariidae)
Source: Data Brief. 2015 Oct 22;5:513–5. doi: 10.1016/j.dib.2015.09.010 (PMC4631843; doi:10.1016/j.dib.2015.09.010)
Supplement: Supplementary file 5 — Supplementary material [file mmc5.pdf]

Ancistrus sp. #1 - GI: KP960569

POSITION	A	C	G	T	Gene	Codon position
2124	200	1	173	946	16S rRNA	n.a.
4297	1747	220	2	0	Nad2	1
5291	1	91	0	444	intergenic	n.a.
7064	72	13	205	623	tRNAs2	n.a.
7858	13	497	72	20	Cox2	1
9556	34	5	41	152	Cox3	2
9558	111	2	29	3	Cox3	1
13581	45	0	397	1	Nad5	2
13586	78	431	0	0	Nad5	1
15458	101	0	12	0	Cob	3

Ancistrus sp. #2a - GI: KP960568

POSITION	A	C	G	T	Gene	Codon position
656	1239	257	0	1	12S rRNA	n.a.
667	0	233	1	1465	12S rRNA	n.a.
2125	103	2	49	465	16S rRNA	n.a.
4298	2452	363	4	0	Nad2	1
7066	45	5	82	595	tRNAs2	n.a.
7072	248	0	37	2	tRNAs2	n.a.
7860	13	495	85	15	Cox2	1
9558	25	2	28	82	Cox3	2

Ancistrus sp. #2b- GI: KP960567

POSITION	A	C	G	T	Gene	Codon position
656	927	208	3	0	12S rRNA	n.a.
667	0	181	1	1113	12S rRNA	n.a.
2125	263	1	53	452	16S rRNA	n.a.
4298	2722	409	6	3	Nad2	1
5292	0	60	0	335	intergenic	n.a.
7072	150	0	21	0	tRNAs2	n.a.
