# Supplementary material for: Mitochondrial transcripts and associated heteroplasmies of Ancistrus spp. (Siluriformes: Loricariidae)
Source: Data Brief. 2015 Oct 22;5:513–5. doi: 10.1016/j.dib.2015.09.010 (PMC4631843; doi:10.1016/j.dib.2015.09.010)
Supplement: Supplementary file 6 — Supplementary material [file mmc6.pdf]

	Protein	5'	ORF	3'	Init. Codon	Term. Codon	"A" add for stop codon	Poly-A length
Ancistrus sp. #1	NAD1	0	972	4	ATG	TAA	0	10
	NAD2	0	1045	0	ATG	TAA	2	11
	COI	1	1551	75	GTG	TAA	0	17
	COII	14	691	0	ATG	TAA	2	16
	ATP8	1	168	673	ATG	TAA	0	0
	ATP6	159	683	0	ATG	TAA	1	9
	COIII	nd	774*	0	nd	TAA	2	17
	NAD3	0	349	0	ATG	TAA	2	10
	NAD4L	0	297	1374	ATG	TAA	0	0
	NAD4	290	1381	0	ATG	TAA	2	9
	NAD5	0	1827	589	ATG	TAA	0	0
	NAD6	0	522	0	ATG	TAA	0	0
	CYT B	0	1136	0	ATG	TAA	2	12
Ancistrus sp. #2a	NAD1	0	972	4	ATG	TAA	0	10
	NAD2	0	1045	0	ATG	TAA	2	nd
	COI	1	1551	75	GTG	TAA	0	11
	COII	14	691	0	ATG	TAA	2	26
	ATP8	1	168	673	ATG	TAA	0	0
	ATP6	159	683	0	ATG	TAA	1	9
	COIII	nd	774*	0	nd	TAA	2	10
	NAD3	0	349	0	ATG	TAA	2	19
	NAD4L	0	297	1374	ATG	TAA	0	0
	NAD4	290	1381	0	ATG	TAA	2	9
	NAD5	nd	1816*	589	nd	TAA	0	0
	NAD6	0	522	0	ATG	TAA	0	0
	CYT B	0	1136	0	ATG	TAA	2	21
Ancistrus sp. #2b	NAD1	0	972	4	ATG	TAA	0	**
	NAD2	0	1045	0	ATG	TAA	2	16
	COI	1	1551	75	GTG	TAA	0	9
	COII	14	691	0	ATG	TAA	2	20
	ATP8	1	168	673	ATG	TAA	0	0
	ATP6	159	683	0	ATG	TAA	1	**
	COIII	0	784	0	ATG	TAA	2	13
	NAD3	0	349	0	ATG	TAA	2	***
	NAD4L	0	297	1374	ATG	TAA	0	0
	NAD4	290	1381	0	ATG	TAA	2	**
	NAD5	0	1827	589	ATG	TAA	0	0
	NAD6	0	522	0	ATG	TAA	0	0
	CYT B	0	1136	0	ATG	TAA	2	9

nd: not determined; *partial CDS; **poliadenilation site located in the middle of a policistronic transcript
